# The Glycerol-Induced Perfusion-Kinetics of the Cat Ovaries in the Follicular and Luteal Phases of the Cycle

**DOI:** 10.3390/diagnostics13030490

**Published:** 2023-01-29

**Authors:** Alexey A. Selifonov, Andrey S. Rykhlov, Valery V. Tuchin

**Affiliations:** 1 Education and Research Institute of Nanostructures and Biosystems, Saratov State University, Saratov 410012, Russia; 2 Clinic “Veterinary Hospital”, Saratov State University of Genetics, Biotechnology and Engineering Named after N.I. Vavilov, Saratov 410012, Russia; 3 Science Medical Center, Saratov State University, Saratov 410012, Russia

**Keywords:** ovarian tissues, follicular phase, luteal phase, glycerol, tissue water, total transmittance spectra, diffuse reflectance spectra, diffusion coefficient, optical clearing efficiency

## Abstract

The method of immersion optical clearing reduces light scattering in tissues, which improves the use of optical technologies in the practice of clinicians. In this work, we studied the optical and molecular diffusion properties of cat ovarian tissues in the follicular (F-ph) and luteal (L-ph) phases under the influence of glycerol using reflectance spectroscopy in a broad wavelength range from 200 to 800 nm. It was found that the reflectance and transmittance of the ovaries are significantly lower in the range from 200 to 600 nm than for longer wavelengths from 600 to 800 nm, and the efficiency of optical clearing is much lower for the ovaries in the luteal phase compared to the follicular phase. For shorter wavelengths, the following tissue transparency windows were observed: centered at 350 nm and wide (46 ± 5) nm, centered at 500 nm and wide (25 ± 7) nm for the F-ph state and with a center of 500 nm and a width of (21 ± 6) nm for the L-ph state. Using the free diffusion model, Fick’s law of molecular diffusion and the Bouguer–Beer–Lambert radiation attenuation law, the glycerol/tissue water diffusion coefficient was estimated as *D* = (1.9 ± 0.2)10^−6^ cm^2^/s for ovaries at F-ph state and *D* = (2.4 ± 0.2)10^−6^ cm^2^/s—in L-ph state, and the time of complete dehydration of ovarian samples, 0.8 mm thick, as 22.3 min in F-ph state and 17.7 min in L-ph state. The ability to determine the phase in which the ovaries are stated, follicular or luteal, is also important in cryopreservation, new reproductive technologies and ovarian implantation.

## 1. Introduction

Every year, around the world, there is an increase in the number of diagnosed oncological diseases, including among patients of reproductive age [1]. For example, in the United States, about 70,000 cancer patients under the age of 45 are diagnosed annually [2]. Patients with malignant neoplasms, according to existing modern medical standards, undergo complex chemotherapy and radiation therapy. As a result of such treatment, there is a high probability of partial or complete loss of fertility in women, due to the high cytotoxicity of antitumor treatment [3,4,5,6]. A large group of patients is young women and girls, whose treatment requires bone marrow transplantation, before which alkylating drugs are used in high concentrations, which in most cases leads to sterilization. Currently, it is possible to preserve the reproductive function of women with cancer, with impaired reproductive function or with premature ovarian failure; this is the cryopreservation (freezing) of healthy ovarian tissue with subsequent transplantation or autotransplantation after recovery [7,8,9,10].

One of the most informative and reliable methods for examining ovarian tissue is laparoscopy, which is widely used in gynecology for both diagnostic and surgical purposes [11,12]. The laparoscope is inserted into the abdominal cavity through a small incision, which allows one to directly examine the organs of the small pelvis and abdominal cavity or, by connecting a video camera, transmit the image to the monitor. However, such an image is formed only by light reflection from the surface of the organ under study; the internal structure of the organ is hidden from observation due to strong light scattering by the tissues of this organ. Using the immersion optical clearing of tissue, it is possible to suppress scattering and observe previously hidden pathological changes in tissues for some time [13,14,15,16,17]. Typical times for the optical clearing of the upper layers of the tissue are 20–40 min. It is important that radiocontrast and MRI contrast agents can act as optical clearing agents. This opens the way to multimodal diagnostics and the support of laparoscopic surgery. It should be noted that hysterosalpingography and hysteroscopy [11,12,18] can be combined in one study with the possibility of obtaining high-quality optical images using laparoscopically compatible optical imaging techniques, as radiopaque and MRI agents are good optical clearing agents [13,14,15,16,17]. 

Cryopreservation is a new method that is successfully used in clinical practice. By 2020, about 85 transplantations of cryopreserved ovarian tissue have been performed, and cases of birth of 30 children have been described, although a longer follow-up of patients is required [19,20]. The standard method for cryopreservation of ovarian tissue is slow freezing using a medium with the addition of cryoprotectants: dimethyl sulfoxide (DMSO), ethylene glycol and 1,2-propanediol (PrOH), which are able to penetrate cell membranes and provide their protection. Slow freezing is carried out with liquid nitrogen for several hours [14,21,22]. However, with slow freezing, there is a risk of damage to cells by ice crystals [8,23,24]. Therefore, penetrating cryoprotectants are often used in combination with non-penetrating ones such as sucrose, glycerol, or human serum albumin [22]. They protect cells through dehydration and stabilization of the lipid layer and proteins. The use of glycerol during the cryopreservation of ovarian tissue can be useful for maintaining the viability of follicles, as glycerol causes the dehydration of cells and, when mixed with water, reduces the freezing point (the temperature of ice formation in cells and solutions) and increases the viscosity of aqueous solutions. After preparing the biological material by soaking it in cryogenic liquids, the vitrification procedure is used, which consists of immersing the material in liquid nitrogen. This method effectively preserves the morphology and viability of the follicles [25,26,27]. 

The method of the cryopreservation of ovarian tissue is new and requires further comprehensive study. Many researchers estimate the recovery of ovarian function after cryopreserved tissue transplantation as short-term. Ischemia that occurs in the first hours after transplantation can lead to the death of more than a third of primordial follicles and, therefore, is the main reason for the decrease in the functional activity of the ovary [28]. To restore the reproductive potential, it is extremely important to reduce the time interval of ischemia and accelerate the revascularization of grafts. In this regard, the transplantation of whole ovarian tissue on a vascular pedicle has been proposed as the most acceptable approach compared to the transplantation of the ovarian cortex alone [29,30]. As shown in animals, the transplantation of an intact whole ovary with microsurgical vascular anastomosis, despite the technical complexity of this procedure, is the only solution to the problem, as it provides a direct blood supply to the ovarian tissue after transplantation, minimizing the risk of ischemia. Therefore, the whole ovary cryopreservation method requires development and further research. The determination of the kinetic parameters of ovarian perfusion with cryoprotectants in different phases of the cycle, including the rate of diffusion of glycerol and the flow of tissue water induced by it, as well as changes in the optical properties of ovarian tissue, is a necessary condition for creating personalized clinical protocols for cryopreservation.

The aim of this work was to study the perfusion-kinetic properties of cat ovaries in the follicular (F-ph) and luteal (L-ph) phases of the cycle by optical clearing method under the influence of glycerol and using diffuse reflectance spectroscopy.

## 2. Materials and Methods

### 2.1. The Structure of the Ovaries and the Cycle

**Figure 1:**
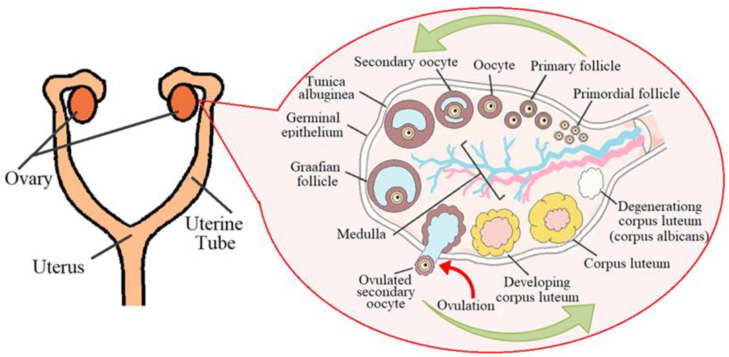
Schematic representation of the ovarian cycle. Adopted from Ref. [31].

The ovaries are a paired organ located on the sides of the uterus, next to the ampullar sections of the fallopian tubes, their size in women ranges from 1.5 to 5 cm [31] and in cats, from 0.5 to 1.5 cm [32]. From above, the ovaries are covered with a layer of the epithelium; the next layer consists of connective tissue and contains many elastic fibers (Figure 1). The medulla contains many blood vessels and nerve endings. In the cortical layer, there are follicles in which eggs are formed and mature.

The female cycle normally lasts from 21 to 35 days. The main phases can be distinguished: 1: follicular (F-ph), in this phase of the cycle, there are many growing and primary follicles in the ovaries (Figure 1); 2: ovulation is a hormone-dependent process of the rupture of the wall of the tertiary (preovulatory) follicle and the release of the female germ cell, and a peak level of hormones is observed: follitropin and lutropin; and 3: luteal phase (L-ph). The corpus luteum has a size of 1.0 to 2.7 cm. A gland of temporary secretion contains lutein and produces a large amount of progesterone, the dominant hormone of the luteal phase, which is important for the safety and proper development of a potential embryo [33].

When studying the blood supply to the ovaries of cows, it was found that the uterine branch of the ovarian artery and especially its anastomosis with the uterine artery were larger on the side of the ovary containing the corpus luteum [34]. The blood supply of the mature corpus luteum is the highest of all body organs per unit volume of tissue. An increase in blood supply is an integral part of the development of the corpus luteum. This important process, mediated by angiogenic growth factors, includes the destruction of the basement membrane of the follicles, the proliferation and migration of endothelial cells, and the development of a large network of capillaries [35]. The corpus luteum functions for only a few (4–7) days and then undergoes involution. A white body (scar) appears in its place [36] (Figure 1). 

### 2.2. Histological Examination

**Figure 2:**
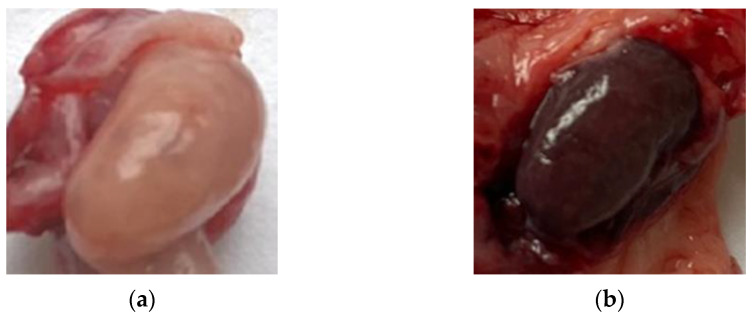
Photos of the studied clinically healthy cat ovaries: (**a**) light; (**b**) dark.

Normal ovarian tissue was taken from outbred cats aged 1 to 12 years with a diagnosis of “clinically healthy”. The ovaries were collected after laparoscopic oophorectomy and ovariohysterectomy from 10 cats. Animals were administered general anesthesia: premedication—meditin (0.1%); intravenous anesthesia—zoletil 100; and alpha 2—antagonist antiemetic for withdrawal from anesthesia. According to visual inspection, all ovaries planned for the study were divided into two groups: “light” and “dark” (Figure 2). Ten light and ten dark tissue samples from different individuals were used for histological examination. 

In vitro histological studies were carried out using halves of each ovary, which were manually cut with a scalpel and fixed. The other halves of the ovaries without fixation were kept frozen for ex vivo optical measurements. The material for histological examination was prepared after no more than 48 h had passed after oophorectomy and ovariohysterectomy; 10% buffered formalin was used for tissue fixation. The thickness of tissue sections was 2–3 μm. The hematoxylin–eosin staining method was used for the histological examination of all samples. 

To obtain histological scans, an Aperio AT2 digital slide converter (on-screen diagnostic scanner) equipped with an LED light source and calibration tools was used. According to the results of the histological studies, it was proved that normal, pathologically unchanged tissues were selected for research, that dark samples contain the corpus luteum and correspond to the luteal phase, and light samples contain multiple follicles, which corresponds to the follicular phase.

### 2.3. Optical Measurements

Measurements of the optical properties of cat ovarian tissue were performed ex vivo without tissue fixation. The thickness of sections (samples) of tissue was measured with an electronic micrometer (Union Source CO., Ltd., Ningbo, China). The measurements were carried out at several points of the sample and averaged. The accuracy of each measurement was ±0.1 mm. The thickness of the tissue section of both dark and light ovaries averaged (0.8 ± 0.1) mm. To measure the diffuse reflectance spectra (DRS) and the total transmittance spectra (TTS) of the tissue samples in the spectral range of 200–800 nm, a Shimadzu UV-2550 double-beam spectrophotometer (Tokyo, Japan) with an integrating sphere was used (Figure 2). A total of 20 ovaries, 10 light and 10 dark, were examined for optical measurements. To study the kinetics of the DRS, ten sections were taken from every five light and five dark samples. Similarly, ten sections from the other five + five ovaries were used to measure the TTS.

**Figure 3:**
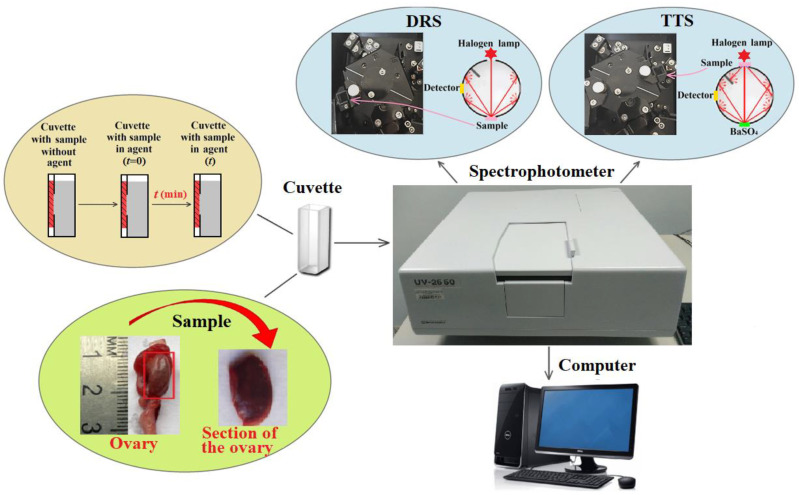
Scheme of the experimental setup for measuring the DRS and TTS of ex vivo samples of cat ovarian tissue.

The radiation source was a halogen lamp with radiation filtering in the studied spectral range. The limiting resolution of the spectrometer was 0.1 nm. Prior to measurements, the spectra were normalized using a BaSO_4_ reference reflector with a suitable reflectivity for the entire spectral range, including UV. All measurements were carried out at room temperature (~25 °C) and normal atmospheric pressure. Each sample of the studied tissue was fixed with a double-sided adhesive tape in a special frame with a window of 0.5 × 0.5 cm in a quartz cuvette so that the tissue sample was pressed against the wall of the cuvette and subjected to optical measurement of DRS or TTS as shown in Figure 3. To measure the TTS, a quartz cuvette with a tissue sample was placed directly in front of the integrating sphere, collecting all the radiation transmitted through the tissue sample. The diameter of the light beam incident on the sample was 3 mm. The initial DRS or TTS spectrum was taken from the ovarian tissue sample pressed against the cuvette wall. Then, glycerol was added into the space between the sample surface and the cuvette wall, after which, measurements were carried out for 100 min until the time dependence was saturated due to the completion of the glycerol/interstitial water diffusion process. The measurement of the DRS kinetics was used for the determination of the diffusion coefficient of the molecular flux induced by the topical application of glycerol to a tissue sample. In the study, a chemically pure 99.5%-glycerol was used (Akrihimfarm LLC., Moscow, Russia). 

It is important to note that, in the wavelength range from 150 to 800 nm, the absorption of glycerol is negligible [37]. However, to determine the effectiveness of optical clearing after the clearing process was completed, the frame with the sample was transferred to a similar, but dry, clean cuvette. Then, the final values of the DRS and TTS of the sample were measured and compared with the initial values before clearing, which were also obtained in a cuvette without glycerol.

## 3. Calculations

**Figure 4:**
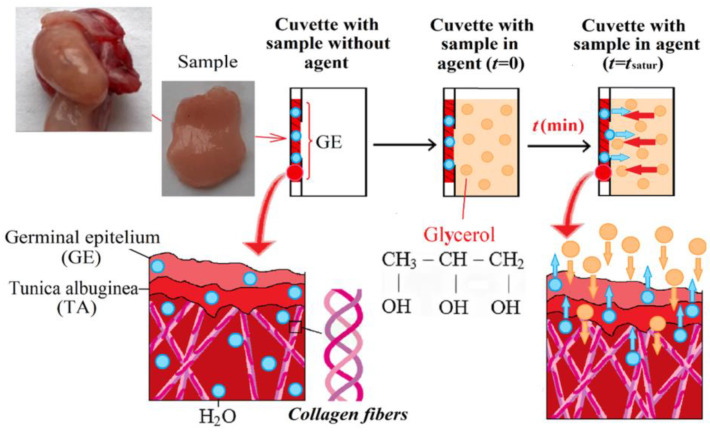
Diagram showing the interaction of a hyperosmotic agent (glycerol) with ovarian tissue.

The determination of the diffusion coefficient of glycerol/interstitial water in tissue is based on measuring the kinetics of the DRS. Figure 4 illustrates this schematically. The process of glycerol/interstitial water transport in a sample can be described in terms of the model of free diffusion [13,14,15,16,17,38,39,40]. Geometrically, a sample of tissue can be represented as a plane-parallel plate of finite thickness. Using the second Fick’s law and performing transformations based on the use of the modified Bouguer–Beer–Lambert law, described in detail in refs. [16,38], we obtain an expression for the difference $\Delta A\left(t,\;\lambda \right)$ between the effective optical density at the current time *A*(*t*, λ) and at the initial time *A*(*t* = 0, λ):

(1) $$\Delta A\left(t,\;\lambda \right)=A\left(t,\;\lambda \right)-A\left(t=0,\;\lambda \right)=\Delta \mu_{\mathrm{eff}}\left(t,\;\lambda \right)L\;\sim \;C_{0}\left\{1-\exp\left(-t/\tau \right)\right\}L$$

$$I=I_{0}\exp\left[-\mu_{\mathrm{eff}}L\right],{\;\mu }_{\mathrm{eff}}\left(t,\;\lambda \right)=\sqrt{3\mu_{a}\left(\mu_{a}+\mu_{s}^{\prime }\right)}\to \Delta \mu_{\mathrm{eff}}\left(t,\;\lambda \right)$$

where the effective optical density is determined from the measurements of DRS:

(2) $$A=-\log R\left(t,\;\lambda \right),$$

(3) $$\tau =\frac{4l^{2}}{\pi^{2}D},$$

*t* is the time in seconds during which the diffusion occurs, λ is the wavelength in nm, ∆μ_eff_ (*t*, λ) is the difference between the effective coefficient of attenuation of light in the tissue $\mu_{\mathrm{eff}}\left(t,\;\lambda \right)$ at the current time and at the initial time, 1/cm; *L* is the average path length of photons, which in the backscattering mode is *L* ≅ 2*l*_d_, (*l*_d_)^−1^ = μ_eff_; µ’_s_ = µ_s_(1 − *g*), 1/cm; *g* is the scattering anisotropy factor (varies from 0 to 1, for many tissues, *g* ≅ 0.93) [16,39]; and for transmission mode *L* ≅ *l*, *l* is the thickness of the sample, cm; *D* is the diffusion coefficient of the glycerol/interstitial water molecules, cm^2^/s; and C_0_ is the initial concentration of the glycerol, mol/L. 

The recorded DRSs [*R*(λ), %] are converted using the standard Kubelka–Munk algorithm to *A*(λ) extinction spectra (Shimadzu UV-2550 spectrophotometer software). 

Glycerol is a well-known effective hyperosmotic agent and is often used for the optical clearing of tissues [13,14,15,16,17,38,39,41,42]. To evaluate the efficiency of optical clearing of ex vivo tissue samples, TTS measurements are usually used, and the efficiency parameter Q is calculated:

(4) Q (%)={T(t,λ)−T(t=0, λ)}/T(t=0, λ),

where *T*(*t* = 0, λ) is the transmittance of the tissue sample for a specific wavelength λ at the initial time, and *T*(*t*, λ) is the same at the current time. 

The bars on the DRS and TTS charts represent the boundaries of the confidence interval, found as:

(5) $$\sigma =\left(t_{s}\mathrm{SD}\right)/\left(\sqrt{n}\right)$$

where *t*_s_ is Student’s coefficient; SD, standard deviation, *n* = 5, *p* = 0.95.

## 4. Results and Discussion

### 4.1. Histological Examination

**Figure 5:**
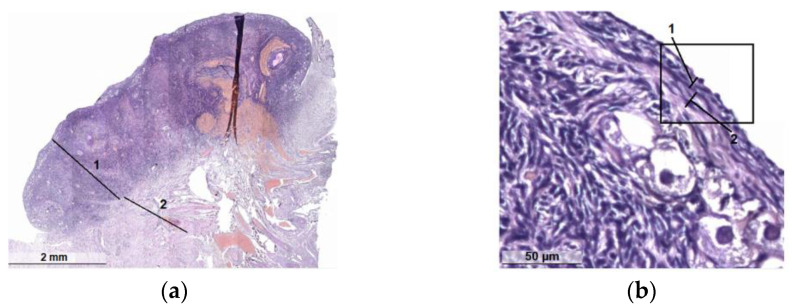
Histology of clinically healthy cat ovaries: (**a**) cortex (1) and medulla (2) in the light ovary (follicular phase); (**b**) the structure of the membrane of the dark ovary (luteal phase): single layer cuboidal epithelium (1) and subepithelial albuginea (2).

Histological examination of a tissue sample from the light ovaries revealed cortical and medulla (Figure 5a); an ovarian capsule was also found for the dark ovaries, including the germinal epithelium (single-layered cuboidal epithelium) and the tunica albuginea (subepithelium) (Figure 5b). However, these structural elements are present in both phases.

**Figure 6:**
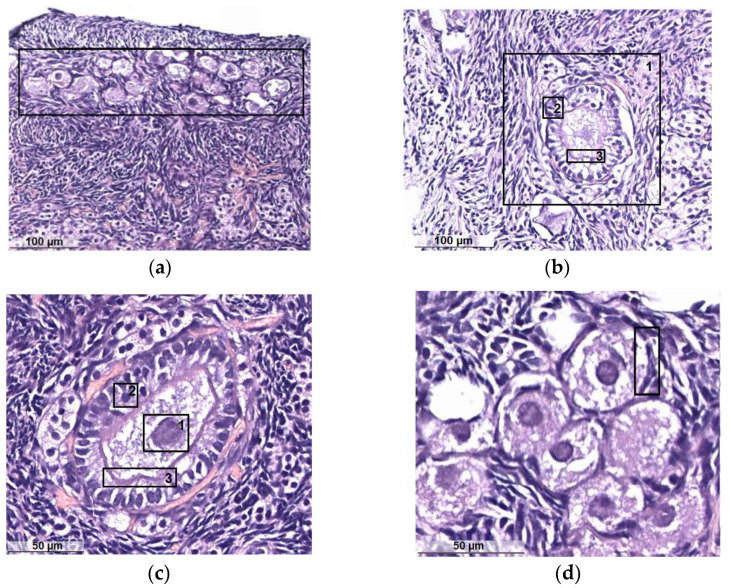
Histology of clinically healthy cat ovaries in the follicular phase: (**a**) primordial follicles; (**b**) primary follicle (1): granulosa of the follicle (2), shiny sheath of the follicle (3); (**c**) oocyte (1): granulosa of the oocyte (2), the shiny coat of the oocyte (3); (**d**) flattened follicular cells surrounding the oocyte.

In the structure of the ovaries, a clear division into the medullary vascular, fibrous cortical layers is determined. In the latter, numerous groups of primordial follicles are noted subcapsularly (Figure 6a) with nearby primary and secondary follicles. Primordial follicles consist of a primary oocyte surrounded by a single layer of flattened follicular cells. Primary follicles include a larger oocyte and a layer or layers of cuboidal granulosa cells and the shiny sheath of the follicle formed around it (Figure 6b). Secondary follicles have a cavity that separates the oocyte with adjacent granulosa cells (crown radiata) from the layers of granulosa cells lining the follicle from the inside of the basement membrane. Outside of such follicles, layers of theca cells are poorly visualized (Figure 6c). The follicles of the cortical layer are located in hypercellular lean fibrous connective tissue, among which, there are single scar-like structures (Figure 6d).

**Figure 7:**
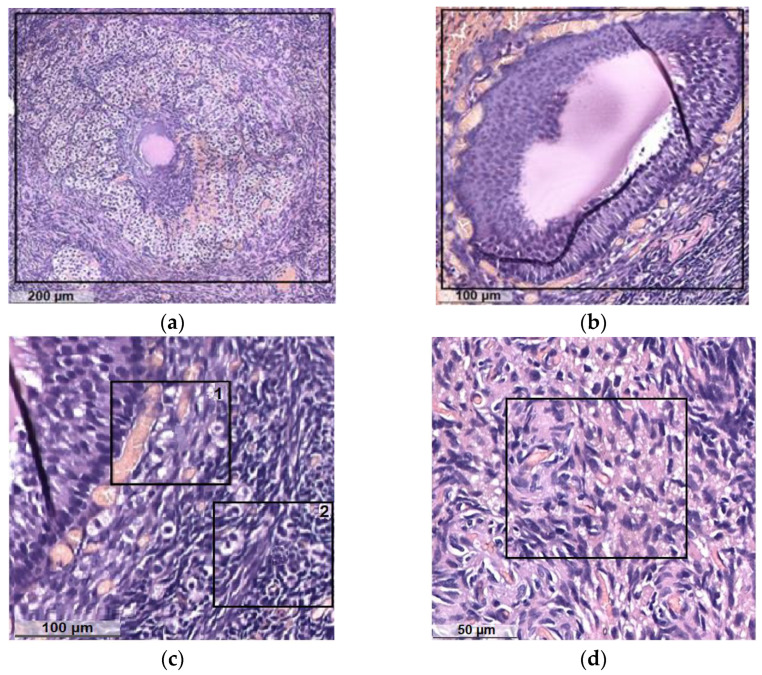
Histology of clinically healthy cat ovaries in the luteal phase: (**a**) corpus luteum; (**b**) atretic follicle; (**c**) follicular theca with an abundance of blood vessels: 1—internal, 2—external; (**d**) interstitial connective tissue.

In dark specimens, the cortical substance is well developed and dominates over the stroma, and the corpus luteum is well visualized (Figure 7a). Some specimens show corpus luteum hyperplasia without atypia. The stroma is represented by a typical theca tissue without edema. It is moderately developed, the capillary network is multiple and small-focal fresh hemorrhages are noted, in some samples multiple. Vessels are thick-walled and unevenly plethoric in some samples with perivascular fibrosis and hyalinosis. Scattered atretic follicles are visualized (Figure 7b) with internal and external follicular theca with an abundance of blood vessels (Figure 7c) and interstitial connective tissue (Figure 7d).

The results of histological studies made it possible to conclude that all the samples of light and dark ovaries taken to study the optical and molecular diffusion properties under the action of glycerol can be attributed to clinically healthy. It was found that light ovaries belong to the follicular phase, and dark ovaries belong to the luteal phase, as the corpus luteum is clearly visualized. Most of the samples showed that the ovaries have a histologically typical structure; in two samples, the histological picture of the ovary with involutive changes was revealed. Necrotic foci, inflammatory infiltrate and atypical growth were not found for all the studied material.

### 4.2. Spectrophotometric Studies

**Figure 8:**
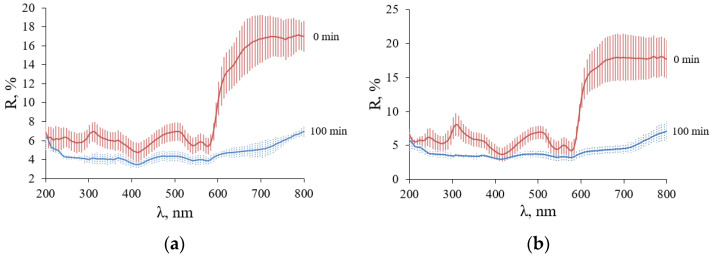
DRC spectra in the range from 200 to 800 nm of cat ovary tissue before and after immersion in 99.5% glycerol during 100 min: (**a**) F-ph (light ovarian tissue); (**b**) L-ph (dark ovarian tissue).

DRSs of the studied samples of cat ovaries in the follicular phase (light ovarian tissue) (F-ph) and in the luteal phase (dark ovarian tissue) (L-ph), initially and after interaction with glycerol, averaged for five samples of each phase of cat ovaries, are shown in Figure 8a,b. It can be seen that the DRSs of both types of samples are almost identical both before and after the diffusion of glycerol. In the UV range, the initial DRSs of the ovarian samples have obvious dips characteristic of the absorption bands of amino acid residues of connective tissue proteins in the form of collagen and reticular fibers, hemoglobin, and porphyrins. In the region of about 415–420 nm and 540–580 nm, the observed dips correspond to the absorption bands of oxyhemoglobin (415, 542 and 576 nm). Water absorption in the measured range of 200–800 nm is insignificant. The main absorption bands in the UV range for common tissue components are located at: 200 nm (proteins), 260 nm (DNA and RNA) and 375 nm (Hb) [16,39]. As a result, tissues are very opaque in the UV range due to the absorption and very strong scattering of light.

**Figure 9:**
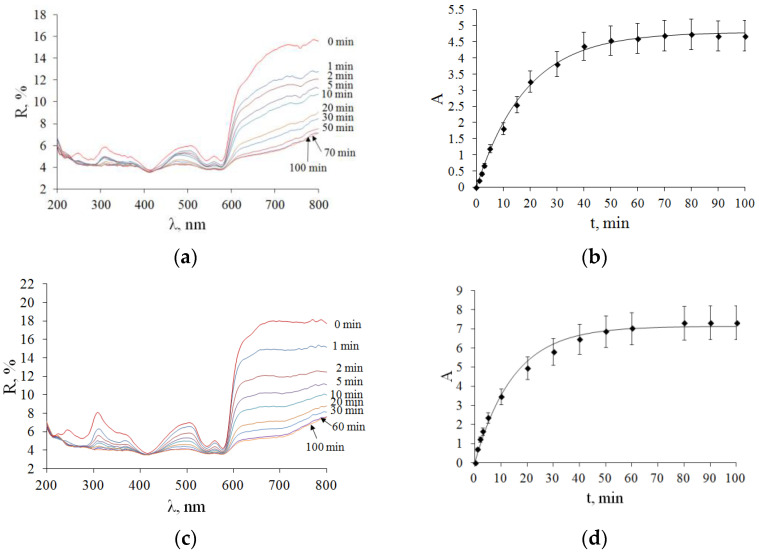
DRSs of cat ovarian tissue samples during 99.5% glycerol immersion. The corresponding kinetics of the difference in effective optical densities at the current and initial time ∆*A*(*t*, λ) were recorded at 600, 700 and 800 nm and then averaged (see Equation (1)) of the studied ovarian samples during the application of glycerol. The symbols represent the experimental data, and the solid curves represent the corresponding approximation of the experimental data within the framework of the free diffusion model; (**a**,**b**) F-ph; (**c**,**d**) L-ph.

The diffusion coefficient of glycerol/interstitial water in the samples was determined from a least squares analysis of a section of the experimental curve characterizing the change in optical density from the time of glycerol action at selected wavelengths. Figure 9a shows the kinetics of DRS during glycerol interaction for 100 min for one of the light samples of cat ovaries. Calculations for each sample were performed for three wavelengths at 600, 700 and 800 nm (Figure 9b).

Figure 9c shows the kinetics of DRSs with glycerol action for 100 min on one of the dark cat ovary samples. In both types of tissues (F-ph) and (L-ph) under study, the slowing down and termination of the diffusion process occurred within about 30 min. It can be seen that the interaction of glycerol with the samples leads to a gradual decrease in the reflectance over the entire wavelength range under study. As highly concentrated glycerol was used, it can be assumed that the main outflow of interstitial water from the sample and, consequently, the dehydration of tissues due to the release of water from the sample determine the temporal behavior of the DRS, which indicates a decrease in light scattering and, accordingly, makes it possible to unambiguously relate the rate of diffusion of water molecules to the rate of change in the DRS.

Using Equation (1), we find τ (diffusion time), which was 22.3 ± 0.6 min for a light sample of ovarian tissue (F-ph), and 17.7 ± 0.7 min for a dark sample of ovarian tissue (L-ph). The average diffusion coefficient for ovarian samples (*n* = 5) in F-ph was *D* = (1.9 ± 0.2)·10^−6^ cm^2^/s, and in the L-ph *D* = (2.4 ± 0.2)·10^−6^ cm^2^/s. The diffusion coefficients of glycerol/interstitial water fluxes in the studied samples determined from the experimental data (Figure 7) according to Equations (1)–(3) and the least squares method are presented in Table 1.

The data obtained can be compared with the values of the diffusion coefficients of molecular flows, which are caused by the topical application of highly concentrated glycerol. The molecular diffusion coefficient measured in human gingival tissue at the action of 99.5% glycerol was found as (1.78 ± 0.22) × 10^−6^ cm^2^/s (*n* = 5; *l* = 0.59 ± 0.06 mm) [41,42], which correlates well with the data received in this paper (Table 1) and the literature data for other tissues [13,14,15,16,17,38,39,40,41,42] taking into account the structural features of tissues and mostly related to tissue water diffusion due to osmotic pressure. As we assume that under the influence of glycerol mainly water migrates in the tissue, the upper limit for the diffusion coefficient should be the rate of water diffusion in the tissue. Based on data for water self-diffusion (*D*_w_ = 3 × 10^−5^ cm^2^/s [43]), and considering that soft tissues contain up to 75% water, we can estimate the rate of water diffusion in a typical tissue, taking into account the effect of hidden diffusion, which is quantified by the ratio of the path length of the molecular flow between two points in a tissue to the direct distance between these points, named tortuosity [17,44]:

(6) Tortuosity=DwD

The tortuosity was estimated at 3.9 for the gingival lamina propria (LP) layer [44] and at 3.5 for the skin dermis [17], which allows to obtain *D*_LP_ = 1.9 × 10^−6^ cm^2^/s and *D*_dermis_ = 2.4 × 10^−6^ cm^2^/s that is in excellent agreement with the measured values of the diffusion coefficient for light ovarian (F-ph) and dark ovarian (L-ph) tissues, with the tortuosity of 3.9 and 3.5, respectively.

Of course, in reality, there is not one flux, but two opposite fluxes: water flux from the tissue and glycerol flux into the tissue. However, at a high concentration of glycerol, the rate of its diffusion in water is low [45]. The diffusion coefficient of glycerol in water at its mass fraction of 84% at room temperature is 2 × 10^−7^ cm^2^/s, which is an order of magnitude lower than the diffusion rate that we obtained experimentally in this study and which is in good agreement with the water diffusion model in the tissue accounting for the phenomenon of tortuosity.

The diffusion time (τ, min) found from the experimental data for the dark ovary in L-ph turned out to be shorter than for the F-ph (light) samples. This may be because L-ph ovaries contain a larger network of capillaries and are therefore more porous and permeable to migrating molecules. Taking into account the thickness of the whole ovary of *l* = 5 mm and using experimentally determined diffusion coefficients from Table 1 and equation (3), we calculated the dehydration time *t*_deh_ of the whole ovary under the action of highly concentrated glycerol. The ovary in the luteal phase is dehydrated after (2.1 ± 0.1) hours, and in the follicular phase, a little longer—after (2.3 ± 0.1) hours. 

**Figure 10:**
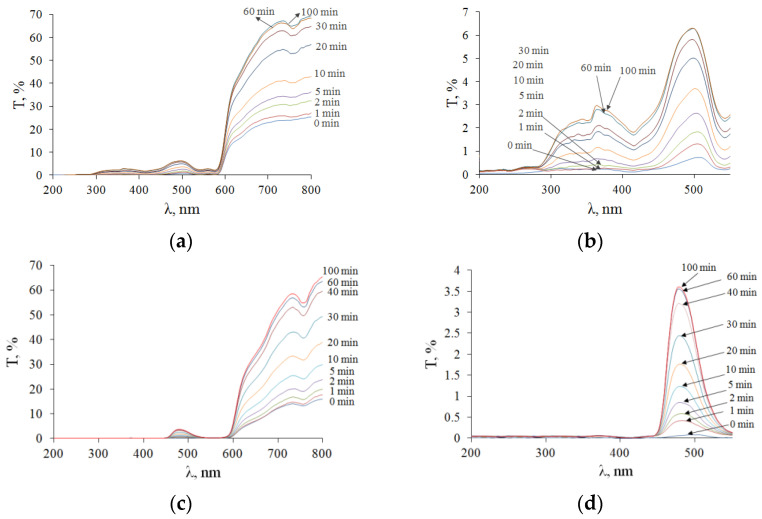
TTSs of cat ovarian tissue samples during 99.5% glycerol immersion: (**a**,**b**) F-ph; (**c**,**d**) L-ph.

**Figure 11:**
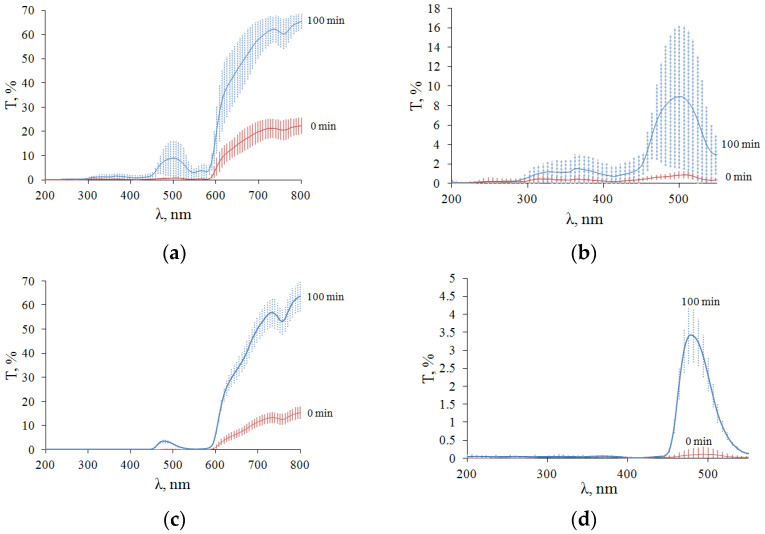
Averaged TTS of 5 samples of cat ovarian tissue sections when immersed in 99.5% glycerol: (**a**,**b**) F-ph; (**c**,**d**) L-ph.

The TTS kinetics for typical samples is shown in Figure 10. In contrast to the DRS, the TTS of the two types of ovarian tissue samples have noticeable differences. The transmittance of both types of samples in the UV is close to zero. The TTSs of light samples (F-ph) show absorption bands of blood hemoglobin, which correlate with the DRSs (Figure 9a). For the TTSs of the dark samples (L-ph), the transmittance is practically zero from 200 to 450 nm and then at 540–590 nm. Obviously, this is due to the fact that this type of ovarian tissue is largely supplied with a capillary network filled with blood. The initial (0 min) and final (100 min) average TTS for all five samples for each type of ovary are shown in Figure 11. 

After the complete immersion of the samples, it can be seen that the optical clearing of tissues occurred with the formation of transparency windows. In samples of ovaries in F-ph, the formation of two transparency windows is observed: one in the UV region with a center 350 nm wide (46 ± 5) nm and with a center 500 nm wide (25 ± 7) nm (Figure 11a,b). In the ovary sample in L-ph, the formation of one transparency window in the visible region of the spectrum with a center of 500 and a width of (21 ± 6) nm is observed, and the UV region does not become more transparent (Figure 11c,d).

The method of immersion optical clearing using hyperosmotic agents, in particular, highly concentrated glycerol, is based on the following mechanisms for suppressing light scattering in tissues. Glycerol induces a partial exchange of tissue water in the interstitial fluid and in the cell cytoplasm and causes tissue dehydration, which, in turn, leads to the matching of the refractive indices of scatterers with the environment (interstitial fluid) and their better packing [13,14,15,16,17,38,39,41,42]. Glycerol has a higher refractive index than interstitial fluid, so when it penetrates the tissues, it also provides refractive index matching, which also causes a decrease in light scattering. As the concentration of glycerol in the tissue becomes sufficiently high, a third mechanism associated with protein dissociation arises [46,47]. However, it is well known that all these mechanisms are reversible and are important for different stages of the optical clearing process [13,14,15,16,17,44,46,47,48,49,50]. 

**Figure 12:**
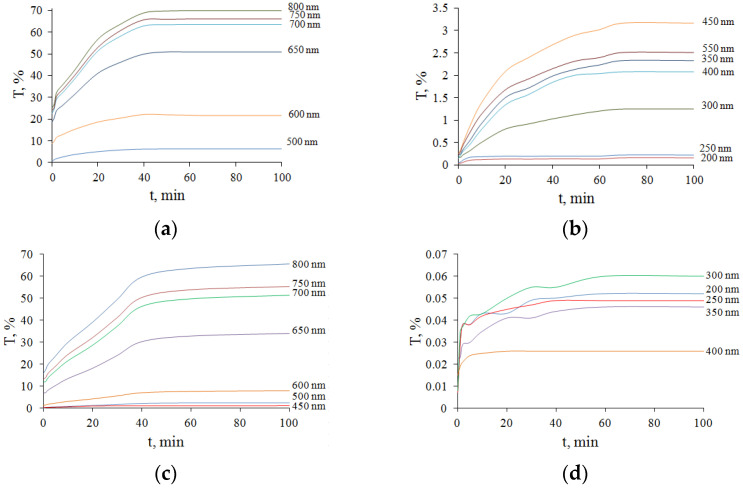
The kinetics of the total optical transmittance *T*(*t*) of cat ovarian tissue samples for different wavelengths λ when exposed to 99.5% glycerol: (**a**,**b**) F-ph; (**c**,**d**) L-ph.

The kinetics of change in transmittance for different wavelengths are shown in Figure 12. When a more blood-filled ovary in the luteal phase interacts with glycerol, hemoglobin is rapidly washed out, which goes quickly, as the erythrocytes burst due to osmosis and, together with tissue water, hemoglobin goes into a larger volume of the surrounding solution. Thus, transmission is increased not only by decreasing scattering but also by decreasing the absorption of hemoglobin and its forms, which is a much faster process (see Figure 12d) [50].

The efficiency of optical clearing under the influence of 99.5% glycerol (*Q*, %), determined by Equation (4), was calculated using experimental data presented in Figure 9a–d for ovarian tissue in different phases of the cycle (Table 2). 

**Figure 13:**
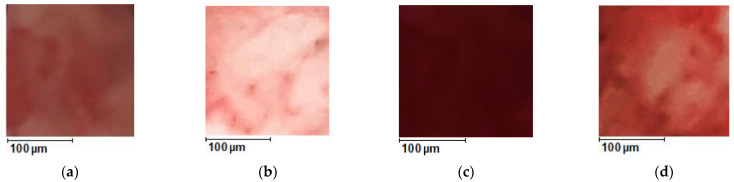
Photos of the area of the samples of the cat ovaries, magnified 50 times: F-ph (**a**) before optical clearing; (**b**) after optical clearing; L-ph (**c**) before optical clearing; (**d**) after optical clearing. The optical clearing agent is 99.5% glycerol.

Figure 13 shows visual changes in the studied tissue samples before and after optical clearing with 99.5% glycerol. The images were taken using the camera of a Samsung Galaxy A51 smartphone with a resolution of 48 MP. To obtain the photos, the samples were placed on a sheet of white paper with white light falling from above.

In the UV range, the efficiency of optical clearing of cat ovarian tissue with 99.5%-glycerol is high and reaches 370% for the F-ph samples and 411% for the L-ph samples. The absolute optical transmittance is not high and reaches 3.5% at 350 nm for F-ph samples (Figure 10b) and only 0.2% for L-ph samples because of strong light scattering combined with strong absorption by the endogenous chromophores, including blood hemoglobin. At 500 nm, the optical clearing efficiency reaches 946% for the F-ph samples and 2074% for the L-ph samples, with the total transmittance up to 16% for the F-ph samples and 4% for the L-ph samples. At 600 nm, the optical clearing efficiency reaches 306% for the F-ph samples and 529% for the L-ph samples, with the total transmittance up to 5% for the F-ph samples and 0.3% for the L-ph samples.

In the so-called “first therapeutic/diagnostic window” at 650–800 nm [16], the efficiency of optical clearing is not the highest and reaches 213% for the F-ph samples and 405% for the L-ph samples. However, due to the absence of strong absorption bands of endogenous chromophores in this region, the absolute transmittance is quite large and amounts to 70% (Figure 10a,c).

Similar results were obtained when using highly concentrated glycerol for the optical clearing of colorectal tissues in normal conditions and in polyposis pathologies, as well as healthy gingival tissue [44]. For the colonic mucosa, two windows of dynamic transparency were identified in the UV range from 200 to 260 nm and from 260 to 418 nm, and a lower efficiency of optical clearing was shown in the long-wave visible/NIR region with a high level of absolute transmittance.

## 5. Conclusions

Two groups of cat ovarian samples were studied. The histological examination of these samples revealed a difference between these groups. It was determined that the light ovaries are in the follicular phase and do not contain a corpus luteum. In the darker ovaries, corpora luteal of various stages were found, which corresponds to the luteal phase of the cycle. The diffuse reflectance and total transmittance of samples in the pre-luteal and luteal phases of the cycle were determined by diffuse spectroscopy. Using the optical kinetics of ovarian tissue samples at glycerol action, glycerol/tissue water diffusion coefficient was estimated, *D* = (1.9 ± 0.2) × 10^−6^ cm^2^/s for ovaries in the follicular stage of the cycle and *D* = (2.4 ± 0.2) × 10^−6^ cm^2^/s for ovaries in the luteal phase of the cycle. Using the obtained diffusion coefficients, it was possible to obtain the time for the complete dehydration of the whole ovary at glycerol action. The time of the complete dehydration of the ovary sections 0.8 mm-thick in the follicular phase was estimated as 22.3 min, and in the luteal phase, 17.7 min. These data can be used to evaluate total ovarian dehydration at concentrated glycerol applications. In general, the data received in this study can be used for designing the protocols for drug delivery and the cryopreservation of organs. 

The total optical transmittance of the ovaries in the follicular phase is much higher than in the luteal phase, which is associated with an anastomosis of an extensive network of capillaries and abundant blood supply to the ovary during this phase of the cycle. Thus, using diffuse spectroscopy, it is possible to fix a fairly short period of formation of the corpus luteum, which is an extremely dynamic temporary organ—a gland that produces progesterone and plays a central role in the reproductive process. The emergence and development of the corpus luteum are extremely rapid with a high cell turnover and a strong blood supply that is primarily regulated by angiogenic growth factors. This ability to accurately determine the timing of lutein formation is extremely important in the study of infertility of unknown origin and when using assisted reproductive technologies.

Optical clearing technology using hyperosmotic agents, in particular glycerol, reduces light scattering and, as a result, improves the penetration depth of light. When ovarian tissue was immersed in glycerol, the efficiency of optical clearing reached 370% in the wavelength range from 280 to 410 nm and up to 946% in the range of 430–550 nm. This effect can be used in therapeutic and diagnostic clinical applications to study molecular structures deep in the tissue. 

The optical clearing technology presented in this study also improves tissue transparency in the UV range and may be useful for the effective application of existing and future UV biomedical spectroscopies and therapies, in particular, to study the structure and dynamics of proteins using UV resonance Raman spectroscopy [51], for the general use of deep UV Raman spectroscopy [52], the detection of pathologies such as gliomas with UV fluorescence excitation [53], or the use of deep UV fluorescence microscopy in cell biology and tissue histology [54] and for other biomedical optical technologies, where UV excitation is fundamentally important. 

The optical clearing and diffusion–kinetic properties of a number of other, more common cryopreservatives, such as DMSO, ethylene glycol and PrOH, have been studied for muscle and skin tissues [13,14,15,16,48]. It seems important to carry out similar quantitative studies for animal ovaries in the follicular and luteal phases of the cycle.

The studied glycerol-induced perfusion kinetics of ovarian tissues is of great importance both for the development of clinical protocols for optical tissue clearing in laparoscopic diagnostic or surgical applications and for the cryopreservation of ovarian tissues. Moreover, the technology can potentially be used for the optical monitoring of changes in tissue structure during the storage of a cryopreserved organ.

An in-depth interdisciplinary study is needed to reduce side effects and preserve fertility in women with cancer [3,4,5,6,10], including those complicated by diabetes mellitus [55], using new technologies for ovarian cryopreservation, including surgical procedures for ovarian transplantation and new reproductive technologies. The successful cryopreservation and subsequent thawing of the transplanted ovary largely depend on the knowledge of the quantitative characteristics of the perfusion–kinetic processes during the freezing and thawing of the organ.

## Figures and Tables

**Table 1:** Kinetic parameters of molecular diffusion in the sections of cat ovaries of the initial thickness *l* = 0.8 ± 0.1 mm and whole ovary (dehydration time *t*_deh_ calculated using diffusion coefficient) at the application of 99.5% glycerol.

Ovarian Tissue	τ, min (Section)	*D* × 10^6^, cm^2^/s	Tortuosity	*t*_deh_, h (Whole Ovary)
Light (F-ph)	22.3 ± 0.6	1.9 ± 0.2	3.9	2.3 ± 0.1
Dark (L-ph)	17.7 ± 0.7	2.4 ± 0.2	3.5	2.1 ± 0.1

**Table 2:** Efficiency (Q, %) of optical clearing of the ovarian tissue in different phases of the cycle.

Phase	λ, nm	200	250	300	350	400	450	500	550	600	650	700	750	800
	*T* (0 min), %	0.04	0.13	0.22	0.25	0.28	0.26	0.85	0.45	3.23	14.51	19.70	20.62	22.30
F-ph	*T* (100 min), %	0.10	0.22	0.64	1.17	0.94	1.95	8.89	3.15	13.13	45.50	57.81	60.82	65.50
	Q, %	148	69	190	370	236	650	946	600	306	213	193	195	194
	*T* (0 min), %	0.009	0.013	0.018	0.021	0.004	0.021	0.11	0.02	1.19	6.50	11.32	12.61	15.21
L-ph	*T* (100 min), %	0.046	0.047	0.044	0.046	0.012	0.074	2.39	0.11	7.50	32.91	49.54	53.71	63.60
	Q, %	411	261	144	119	200	250	2074	450	529	406	338	326	318

## Data Availability

Data are available from the authors.
